# Sperm TLR2/1 activation modulates the paternal RNA cargo and enhances bovine embryonic development

**DOI:** 10.3389/fphys.2026.1906057

**Published:** 2026-09-23

**Authors:** Alireza Mansouri, Ihshan Akthar, Taiyo Hashimoto, Kazuya Kusama, Kazuhiko Imakawa, Masayuki Shimada, Akio Miyamoto

**Affiliations:** 1 Global Agromedicine Research Center (GAMRC), Obihiro University of Agriculture and Veterinary Medicine, Obihiro, Japan; 2 Department of Endocrine Pharmacology, Tokyo University of Pharmacy and Life Sciences, Tokyo, Japan; 3 Research Institute of Agriculture, Tokai University, Kumamoto, Japan; 4 Graduate School of Integrated Sciences for Life, Hiroshima University, Higashi-Hiroshima, Japan

**Keywords:** cattle IVF, embryo development, *in vitro* embryo production, paternal RNA, TLR2/1

## Abstract

*In vitro* fertilization (IVF) and *in vitro* embryo production (IVP) are crucial technologies for accelerating genetic gain in cattle. However, IVP efficiency remains suboptimal, as *in vitro*-derived embryos frequently exhibit reduced developmental competence compared to their *in vivo* counterparts. Previous studies indicate that activation of the sperm Toll-like receptor 2/1 (TLR2/1) complex *via* Pam3CSK4 (PAM3) enhances sperm-oocyte interactions and improves cleavage and blastocyst rates; however, the underlying molecular mechanisms remain unexplored. In this study, frozen-thawed active sperm from three high-fertility Holstein bulls were allocated to control or PAM3-treated groups before IVF. Both sperm and the resulting day 7 blastocysts were subjected to RNA sequencing (RNA-seq) to investigate whether TLR2/1 activation modulates the paternal RNA cargo and if these alterations persist in the embryonic transcriptome. PAM3 treatment significantly improved developmental outcomes, specifically cleavage and blastocyst rates, although the magnitude of the effect varied among bulls. Transcriptomic analysis revealed distinct bull-specific RNA profiles and demonstrated that PAM3 treatment induced selective remodeling of sperm transcript abundance, involving both coding and non-coding RNA fractions. Conversely, day 7 embryos derived from PAM3-treated sperm did not exhibit global transcriptomic divergence from controls. This suggests that the molecular consequences of sperm TLR2/1 activation may be restricted to earlier developmental stages or regulated through specific pathways rather than broad transcriptomic remodeling at the blastocyst stage. In conclusion, these findings suggest that TLR2/1 signaling modulates paternal RNA cargo, potentially contributing to improved fertilization and bovine IVP efficiency by facilitating sperm penetration of the oocyte.

## 1 Introduction

*In vitro* fertilization (IVF) and *in vitro* embryo production (IVP) are vital technologies in bovine reproduction, facilitating the rapid dissemination of high-value genetics. By utilizing advanced breeding tools, such as ovum pick-up (OPU), sexed semen, and genomic selection, IVP systems significantly increase the number of offspring from superior donors compared with traditional breeding. Consequently, IVP has become an essential component of modern dairy and beef production, with *in vitro*-produced embryos now dominating commercial embryo transfer programs (Ferré et al., 2020). Despite this widespread adoption, IVP efficiency remains suboptimal due to challenges in oocyte maturation, fertilization kinetics, and embryonic developmental competence. Improving these outcomes is therefore essential for maximizing reproductive efficiency and accelerating genetic gain (Lonergan, 2024).

Recent research has identified the Toll-like receptor 2/1 (TLR2/1) pathway as a novel target for enhancing bovine fertilization and subsequent embryonic development (Ma et al., 2022, Ma et al., 2025). TLR2/1 is a heterodimeric innate immune receptor complex that primarily recognizes triacylated lipopeptides (Mansouri et al., 2024, Mansouri et al., 2025). Upon ligand binding, which structurally bridges the TLR2 and TLR1 subunits, the complex initiates a MyD88-dependent signaling cascade. This leads to the activation of key transcriptional regulators, most notably nuclear factor kappa B (NFKB), which modulates the expression of various immune and functional genes (Mansouri et al., 2024, Mansouri et al., 2025).

Beyond its classical role in pathogen recognition, TLR2/1 signaling is a critical mediator of sperm-uterine crosstalk. During insemination, sperm preferentially induce the expression of TLR2 and TLR1, but not TLR6, in bovine endometrial epithelial cells (Mansouri et al., 2023). This activation triggers a controlled, transient pro-inflammatory response that is essential for uterine clearance and the establishment of a receptive environment for embryo implantation (Mansouri et al., 2023). Furthermore, the TLR2/1 pathway has been implicated in regulating sperm function, and inflammatory signaling, as well as modulating uterine responses to semen extenders during artificial insemination (Ezz et al., 2023; Mansouri et al., 2023; Akthar et al., 2024; Hulugalla et al., 2025; Waehama et al., 2025).

In the context of bovine IVF, the pre-fertilization activation of sperm TLR2/1 has been shown to improve cleavage and blastocyst rates (Ma et al., 2025; Hashimoto et al., 2026). Mechanistically, this effect is partially attributed to the modulation of the zona pellucida-triggered acrosome reaction; TLR2/1 stimulation induces an influx of intracellular Ca^2+^, promoting the acrosome reaction and subsequently facilitating oocyte penetration and successful fertilization (Ma et al., 2022). In fact, the data indicate that sperm TLR2/1 activation enhances embryo development by improving metabolic and cellular processes, including autophagy, Ca^2+^ influx, and apoptotic regulation, from the 4-cell and 8-cell stages through to the blastocyst stage (Hashimoto et al., 2026). However, the downstream mechanisms through which transient sperm TLR2/1 activation exerts long-term effects on embryo development remain poorly defined. Because mature sperm possess a transcriptionally silent nucleus, immediate changes in transcript abundance following acute ligand exposure are unlikely to arise from nuclear *de novo* transcription (Grunewald et al., 2005). Instead, such alterations are predominantly driven by post-transcriptional mechanisms or alterations in mitochondrial DNA-derived gene expression, because mitochondrial DNA remains transcriptionally and translationally active to support sperm kinetics and fertilization capacity (Zhu et al., 2019b, Zhu et al., 2019a).

Beyond driving fertilization dynamics, it is now well-established that sperm deliver a complex molecular cargo to the oocyte at fertilization, including specific coding and non-coding RNA fractions that function as epigenetic regulators of early embryogenesis (Barroso et al., 2009; Chen, 2026). Therefore, it is essential to profile the immediate transcriptomic changes within the gamete itself to understand how this paternal legacy is shaped. Consequently, this study aimed to investigate whether TLR2/1 activation *via* Pam3CSK4 (PAM3) alters the sperm RNA profile across individual Holstein bulls, and to determine if these targeted paternal transcriptomic modifications persist or disappear in the global transcriptome of the resulting day 7 blastocysts.

## 2 Materials and methods

### 2.1 Experimental design

To investigate the impact of Pam3CSK4, a synthetic TLR2/1 agonist (ab142085, Abcam; hereafter referred to as PAM3), on the paternal RNA profile and subsequent embryonic development, a two-phase experimental design was employed (**Figure 1**). Frozen-thawed ejaculated sperm from three high-fertility Holstein bulls (Bull 1, Bull 2, and Bull 3) were used. In the first phase, sperm samples were allocated into two groups: a control group and a treatment group. The treatment group was incubated with 100 ng/mL PAM3 for 30 minutes. This specific concentration and exposure time were selected based on our previous investigations, which demonstrated optimal enhancement of fertilization rates and sperm functional parameters under these conditions (Ma et al., 2022; Akthar et al., 2023). Following incubation, total RNA was extracted from both groups for comparative transcriptomic analysis *via* RNA-seq. In the second phase, untreated (control) and PAM3-treated sperm were used for IVF to evaluate paternal carry-over effects. This yielded control embryos (derived from untreated sperm) and PAM3 embryos (derived from PAM3-treated sperm). Developmental competence was monitored by assessing cleavage rates on day 1.5 and blastocyst rates on day 7 post-insemination. Finally, the resulting day 7 blastocysts from both groups were collected for RNA-seq to determine if the paternal transcriptomic modifications induced by TLR2/1 activation were reflected in the global embryo transcriptome.

**Figure 1:**
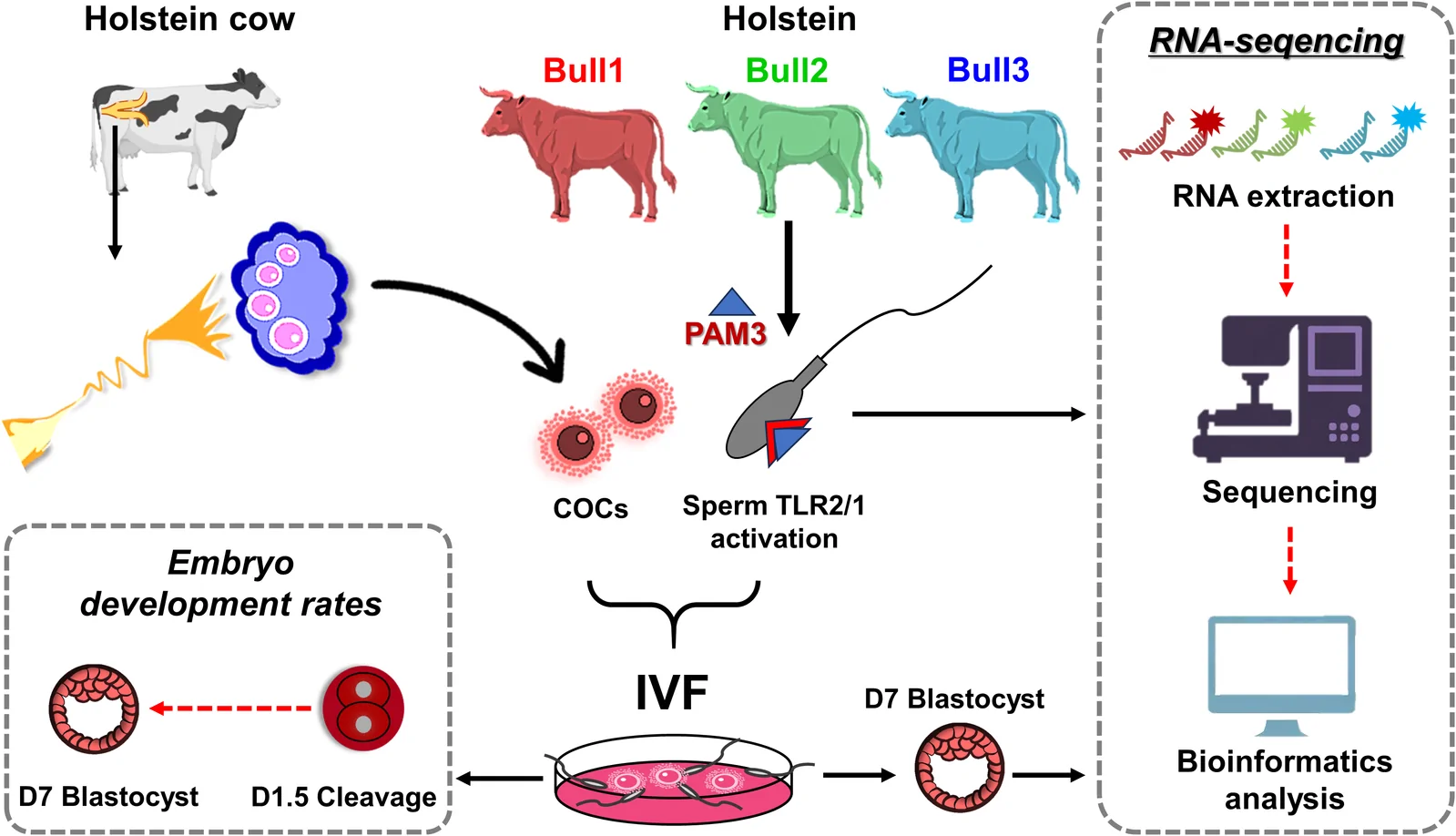
Schematic representation of the research design. Sperm from three high-fertility Holstein bulls were stimulated with 100 ng/mL PAM3, a TLR2/1 agonist, followed by two main procedures: RNA-seq analysis and *in vitro* fertilization (IVF). First, sperm RNA profiles were analyzed to assess the effect of TLR2/1 activation. Second, control and TLR2/1-activated sperm were used for IVF. For IVF, oocytes collected from Holstein cows were co-cultured with either control sperm or PAM3-treated sperm. After IVF, embryo development was evaluated based on cleavage and blastocyst rates in embryos derived from control sperm and PAM3-treated sperm. Finally, Day-7 blastocysts from both groups were subjected to RNA-seq analysis to determine whether sperm TLR2/1 activation altered the embryonic RNA profile.

### 2.2 Sperm sample preparation and TLR2/1 activation

Selective stimulation of the sperm TLR2/1 heterodimer was achieved using PAM3. Based on previously optimized protocols for Holstein sperm, sperm were treated with 100 ng/mL PAM3 for 30 min (Ma et al., 2022; Akthar et al., 2023). Sperm preparation was conducted using a PureCeption (SAGE *In-Vitro* Fertilization, Inc., Trumbull, CT, USA) discontinuous density gradient as previously described, with modifications (Buranaamnuay, 2019). Frozen semen straws from three high-fertility Holstein bulls (designated Bull 1, Bull 2, and Bull 3) were provided by the Genetics Hokkaido Association (Shimizu, Hokkaido, Japan). Three independent biological replicates were performed for each bull. Different semen straws from the same bull were used for each replicate. For each experimental replicate, straws were thawed in a water bath at 37 °C for 30 s. Each semen straw had a volume of 0.5 mL and contained approximately 50 million sperm/mL, corresponding to approximately 25 million sperm per straw. We used 12 straws for each experiment, providing approximately 300 million sperm in total. The thawed semen was carefully layered onto a 40%/80% (v/v) discontinuous PureCeption gradient, consisting of 0.5 mL semen over 2 mL of 40% and 2 mL of 80% gradient media, and centrifuged at 700 × g for 15 min. Following centrifugation, the supernatant was aspirated, and the highly motile sperm pellet was resuspended in sperm Tyrode’s albumin lactate pyruvate (SP-TALP) medium (Akthar et al., 2023). The resulting sperm suspension (40% sperm recovery) was subsequently divided into two experimental groups: a PAM3-treated group (100 ng/mL) and an untreated control group (each treatment group has 60 million sperm cells throughout the treatment). Both groups were incubated at 38.5 °C for 30 min in a humidified atmosphere of 5% CO_2_. Following incubation, the sperm suspensions were washed once in SP-TALP *via* centrifugation at 300 × g for 5 min. The final pellets were resuspended in either lysis mixture containing 0.1 mL of RLT lysis buffer from the RNeasy Plus Mini Kit (Qiagen, USA), 0.9 mL of TRIzol reagent (Invitrogen, Carlsbad, CA, USA), and 0.1 mL of tris(2-carboxyethyl) phosphine hydrochloride (TCEP; Sigma, USA) for total RNA extraction and RNA-seq analysis, or IVF100 fertilization medium (Research Institute for the Functional Peptides, Yamagata, Japan) for IVF.

### 2.3 Sperm RNA extraction for sequencing analysis

Total RNA was extracted from purified sperm samples (50 million sperm cells per biological replicate) using a modified version of a previously established protocol (Umar et al., 2025). Sperm pellets were homogenized and lysed in the lysis mixture (0.1 mL RLT lysis buffer, 0.9 mL TRIzol reagent, 0.1 mL TCEP). To ensure efficient phase separation, 0.2 mL of chloroform was added to the lysate, followed by centrifugation. The aqueous phase was collected, and subsequent purification steps were performed using the RNeasy Plus Mini Kit (Qiagen, USA) according to the manufacturer’s instructions. This kit includes an integrated gDNA Eliminator column, which was supplemented with an on-column DNase I treatment (Qiagen, USA) to ensure the complete removal of genomic DNA contamination. Following extraction, RNA structural integrity was assessed using an Agilent 2100 Bioanalyzer. Consistent with the characteristic profile of mature bovine sperm, the RNA Integrity Number (RIN) for all samples was less than 3.0 (Roszkowski and Mansuy, 2021; Navarrete-López et al., 2024). This fragmentation pattern was interpreted as a representative feature of the sperm transcriptomic landscape, which consists primarily of highly processed and fragmented RNA (Roszkowski and Mansuy, 2021; Navarrete-López et al., 2024). Isolated total RNA was stored at -80°C before library preparation and sequencing.

### 2.4 Collection of cumulus-oocyte complexes and *in vitro* maturation

Collection of COCs and IVM were performed according to previously published methods with slight modifications (Ma et al., 2022, Ma et al., 2025). Briefly, Holstein ovaries were collected from a local slaughterhouse in Obihiro, Hokkaido, Japan, and transported to the laboratory within 1–2 h in physiological saline (0.9% NaCl) containing 1% penicillin-streptomycin (Gibco, Grand Island, NY, USA). COCs were aspirated from follicles measuring 2–8 mm in diameter using an 18-gauge needle connected to a 10 mL syringe. Recovered COCs were washed three times in oocyte collection medium (OCM; Research Institute for the Functional Peptides Co., Ltd., Yamagata, Japan) supplemented with 10% fetal bovine serum (FBS; Biowest, Nuaillé, France). Only COCs with uniform cytoplasm and surrounded by at least three compact layers of cumulus cells were selected for maturation. Groups of 30–60 selected COCs were cultured in high-performance modified 199 medium (HP-M199; Research Institute for the Functional Peptides Co., Ltd.) supplemented with 10 ng/mL epidermal growth factor (EGF; Sigma-Aldrich, St. Louis, MO, USA) and 10% FBS. Maturation was carried out at 38.5 °C for 22 h in a humidified incubator containing 5% CO2 in air.

### 2.5 *In vitro* fertilization and *in vitro* culture

Matured COCs were then inseminated by co-incubation of 10–20 COCs with the prepared sperm suspension (0.2 million/mL) at 38.5 °C for 6 h under 5% CO2 in humidified air. At 6 h post-insemination (hpi), cumulus cells were removed from presumptive zygotes by gentle repeated pipetting. Subsequently, groups of 30–60 zygotes were placed into 4-well culture dishes containing BO-IVC medium (IVF Bioscience, Falenty, Poland) covered with mineral oil. Embryos were cultured at 38.5 °C in a humidified tri-gas incubator maintained at 5% O_2_, 5% CO_2_, and 90% N_2_. Cleavage was assessed at 36 hpi (day 1.5), and blastocyst development was evaluated on day 7.

### 2.6 Embryo RNA extraction for sequencing analysis

Total RNA was extracted from day 7 blastocysts (pools of approximately 20 embryos per biological replicate) using a modified TRIzol-based protocol with minor modifications (Ezz et al., 2023). Embryo morphological quality was assessed according to the International Embryo Technology Society (IETS) guidelines (Stringfellow et al., 1998). Embryos were categorized into two quality groups: Grade A, comprising code 1 (excellent) and code 2 (good) embryos, and Grade B, comprising code 3 (fair) embryos. Code 4 embryos (dead or degenerating) were excluded from the study. This standardized classification ensured that only developmentally competent blastocysts were selected for transcriptomic analysis.

Briefly, pooled embryo samples were lysed and homogenized in TRIzol reagent, followed by phase separation using 100 µL of chloroform. After centrifugation at 12,000 × g for 15 min, 200 µL of the upper aqueous phase was carefully collected and transferred to a new tube. RNA was then precipitated by adding 250 µL of isopropanol, followed by centrifugation at 12,000 × g for 10 min. After removal of the supernatant, the RNA pellet was washed twice with 450 µL of 75% ice-cold ethanol and centrifuged at 12,000 × g for 5 min. The ethanol was then removed, and the RNA pellet was resuspended in RNA storage solution (Ambion, Austin, TX, USA). RNA samples were stored at −80 °C until sequencing. The quantity and structural integrity of the embryonic RNA were evaluated using an Agilent 2100 Bioanalyzer. In contrast to the naturally fragmented profile observed in sperm, the embryo samples exhibited high RIN values (higher than 7.0), confirming high-quality, intact total RNA suitable for transcriptomic profiling *via* RNA-seq (Guzeloglu et al., 2026).

### 2.7 Bioinformatics analysis

#### 2.7.1 Sequencing and quality control

Total RNA samples were first quality-checked (RNA integrity by Agilent 2100 Bioanalyzer) and used to prepare stranded cDNA libraries, which were sequenced on BGI’s DNBSEQ platform (Low input RNASeq with the NuGen Revelo kit) in paired-end 100 bp mode. The raw FASTQ reads were processed with SOAPnuke for quality control and filtering (Chen et al., 2018). SOAPnuke was run with parameters to trim adapters and low-quality bases and to discard reads with high N-content or long poly-X runs. In brief, reads matching ≥25% adapter sequence (allowing up to two mismatches) were removed, reads shorter than 100 bp after trimming were dropped, any read with ≥1% ambiguous bases (N) or with a poly-A/T/G/C stretch >50 bp was discarded, and reads with >40% bases below Q20 were removed. The resulting clean reads (Phred+33) were retained in FASTQ format for downstream analysis. Quality metrics such as per-base quality scores, GC content, read length distribution, and overall sequence duplication were assessed to confirm data suitability.

#### 2.7.2 Read mapping and quantification

Clean paired-end reads were aligned to the *Bos taurus* UMD3.1 reference genome using HISAT2 v2.2.1 (Kim et al., 2015). Gene-level counts were generated using HTSeq-count (Anders et al., 2015). Reads overlapping annotated gene features were assigned to genes, and the resulting count matrix was used for downstream differential transcript abundance analysis. HTSeq-count summary categories, including reads not assigned to annotated features, ambiguous reads, non-aligned reads, and non-unique alignments, were also retained for quality assessment. Mapping and counting quality were evaluated using alignment statistics and read-counting summary information.

#### 2.7.3 Differential expression analysis

Differential expression analysis was performed in R using DESeq2. Briefly, the raw count matrix (genes × samples) was input to DESeq2, which models read counts via a negative-binomial generalized linear model (Love et al., 2014). This approach uses size-factor normalization and dispersion estimation to test for expression differences between defined groups (e.g., treatment vs. control). Genes with a false discovery rate (FDR) below 0.1 were considered significant; typically, a fold-change threshold (|log_2_FC| ≥ 2) would also be applied to focus on biologically meaningful changes (Yamane et al., 2025). The results were visualized with standard plots: volcano plots to display the relationship between fold change and significance, and hierarchical clustering heatmaps of the differentially expressed genes (DEGs) across samples. These visualizations confirmed the overall patterns of increased and decreased transcript abundance among the DEGs.

### 2.8 Statistical analysis

GraphPad Prism 5 software (GraphPad Software, La Jolla, CA, USA) was used for statistical analysis. The data were normally distributed. Student’s t-test was applied to compare the mean differences between two groups. All values are presented as mean ± standard error of the mean (SEM). Data were considered to be statistically significant at *P<0.05.

## 3 Results

### 3.1 Global transcriptomic variation and sample clustering in sperm

Principal component analysis (PCA) and heatmap analysis (Li et al., 2024) were performed to evaluate global gene expression patterns in sperm samples and to examine both inter-bull variability and the effect of PAM3 treatment.

First, PCA was used to compare sperm transcriptomic profiles among the three high-fertility Holstein bulls (**Figure 2A**). PC1 and PC2 explained 45% and 15% of the total variance, respectively, together accounting for 60% of the variation in the dataset. Samples from each bull formed distinct clusters, particularly along PC1, indicating clear inter-bull variation in sperm transcriptomic profiles despite all bulls being classified as highly fertile. To further examine the effect of TLR2/1 activation, PCA was performed separately for control and PAM3-treated samples within each bull (**Figure 2B**). The first two principal components explained 65%, 63%, and 62% of the total variance in Bulls 1, 2, and 3, respectively, indicating that the PCA captured a substantial proportion of the transcriptomic variation within each bull. PAM3 treatment was associated with changes in the clustering pattern of sperm samples in all bulls; however, the pattern of response differed among individuals. In Bull 1, control samples clustered closely together, whereas PAM3-treated samples were more dispersed. In contrast, control samples from Bulls 2 and 3 showed greater dispersion, while PAM3-treated samples clustered more closely, suggesting a more consistent transcriptomic response to PAM3 treatment in these two bulls. These findings suggest that the transcriptomic response to PAM3 is not uniform across bulls, possibly reflecting individual bull-dependent differences in baseline sperm RNA profiles or responsiveness to TLR2/1 activation. Therefore, while PAM3 stimulation affected sperm transcriptomic profiles within each bull, the PCA pattern also highlights the importance of inter-bull variation when interpreting treatment-associated transcriptomic changes. In addition, to assess clustering between the control and PAM3-treated groups, a separate PCA was performed using all control and treated samples from the three bulls (**Supplementary Figure 1**). No clear separation was observed between the control and PAM3-treated groups when the data from all bulls were pooled. If the treatment effect had been consistent across bulls, one cluster for the control samples and another cluster for the PAM3-treated samples would have been expected. However, because of the high variation among bulls, the samples largely overlapped and did not form clear treatment-specific clusters.

**Figure 2:**
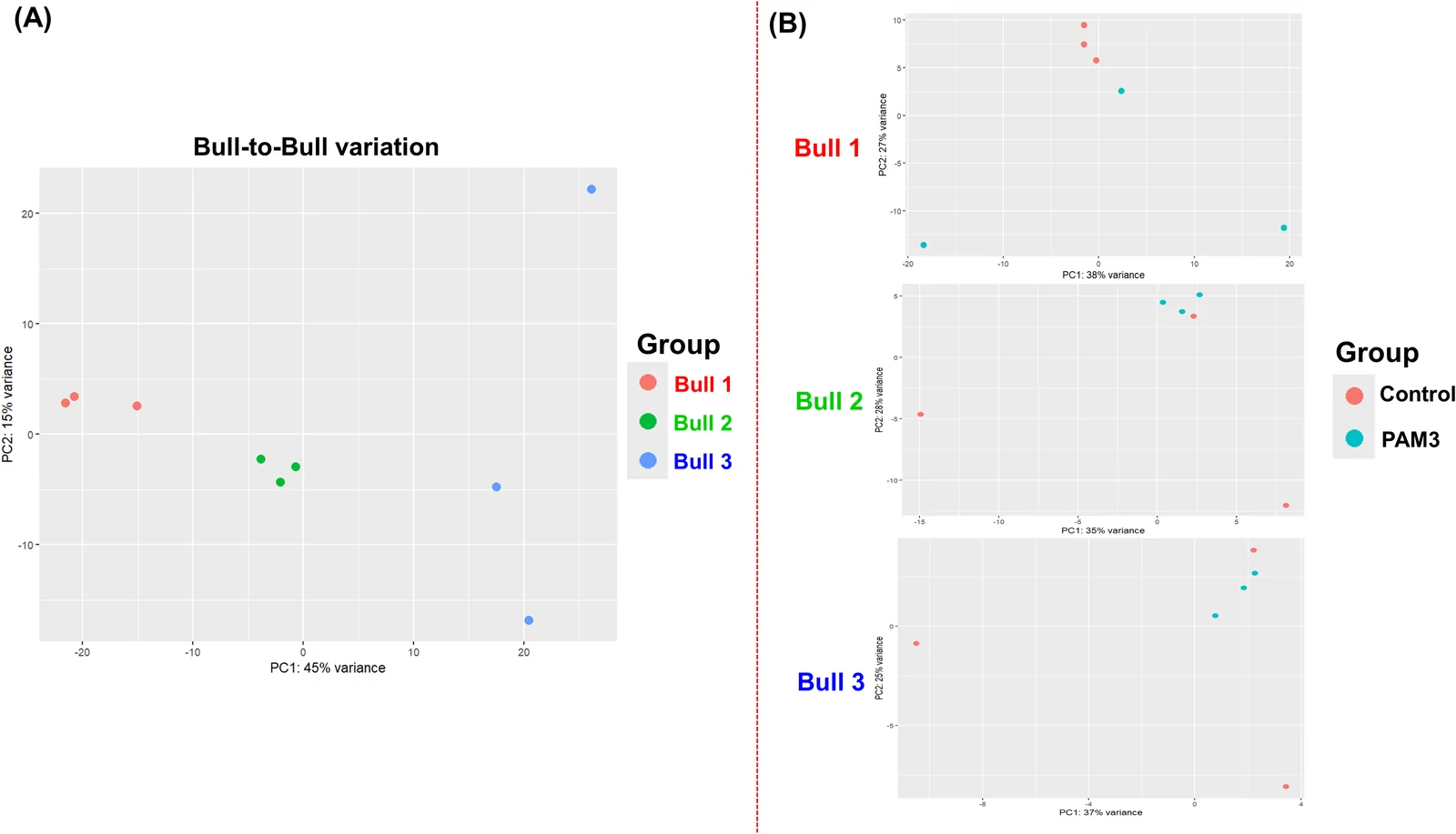
Principal component analysis (PCA) of sperm transcriptomes. **(A)** PCA plot showing transcriptomic variation among sperm samples from three high-fertility Holstein bulls. For comparing three bulls, only the untreated control samples from the three bulls were included. The purpose of this analysis was to assess the intrinsic transcriptomic variation among bulls independently of PAM3 treatment. PC1 and PC2 explained 45% and 15% of the total variance, respectively. Although all bulls were classified as high-fertility bulls, the PCA plot indicates bull-dependent variation in sperm transcriptomic profiles. **(B)** For comparing control and PAM3, the same control samples were subsequently compared with their corresponding PAM3-treated samples in separate within-bull analyses. PCA plot showing the effect of TLR2/1 activation by PAM3 treatment on the sperm transcriptome of each bull. PAM3 treatment altered the transcriptomic distribution of sperm samples in all bulls; however, the pattern of change differed among individual bulls. In Bull 1, control samples clustered closely together, whereas in Bulls 2 and 3, PAM3-treated samples showed tighter clustering. Thus, the PCA suggests that TLR2/1 activation affects the sperm transcriptome in a bull-dependent manner.

Heatmap analysis was then performed using differentially expressed genes identified based on FDR < 0.1 to further examine expression-pattern differences among bulls and between treatment groups (Yamane et al., 2025). Approximately 120 genes were differentially expressed among Bulls 1, 2, and 3, supporting the presence of inter-bull transcriptomic variation even among high-fertility bulls (**Figure 3A**). In the comparison between control and PAM3-treated sperm, the heatmap showed distinct expression patterns, with some genes showing higher abundance in control samples and others showing higher abundance in PAM3 group (**Figure 3B**). Together, the PCA and heatmap results indicate that sperm transcriptomic profiles differ among individual bulls and that PAM3-mediated TLR2/1 activation is associated with changes in the abundance of specific sperm RNA transcripts.

**Figure 3:**
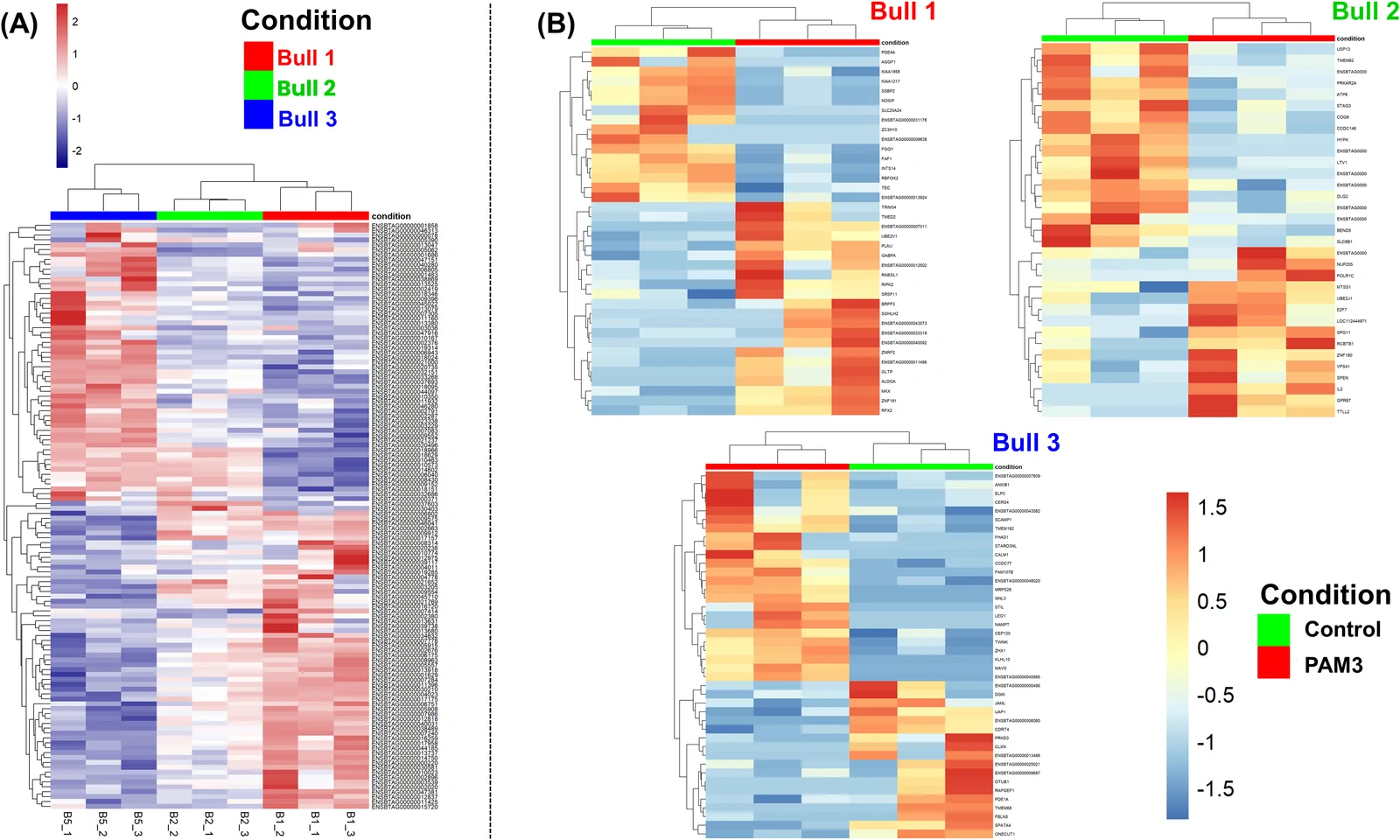
Heatmap analysis of sperm transcriptomes. **(A)** Heatmap showing transcriptomic variation among sperm samples from three high-fertility Holstein bulls. For comparing three bulls, only the untreated control samples from the three bulls were included. The purpose of this analysis was to assess the fundamental transcriptomic variation among bulls independently of PAM3 treatment. Approximately 120 genes showed different expression patterns among the bulls, although all bulls were classified as high-fertility bulls. **(B)** For comparing control and PAM3, the same control samples were subsequently compared with their corresponding PAM3-treated samples in separate within-bull analyses. Heatmap showing the effect of TLR2/1 activation by PAM3 treatment on the sperm transcriptome of each bull. The heatmap indicates that the abundance of several mRNA transcripts was either increased or decreased in PAM3-treated sperm compared with control sperm. Since sperm are transcriptionally inactive, these results suggest that TLR2/1 activation may alter the abundance and degradation of existing sperm transcripts rather than induce new transcription.

### 3.2 Transcriptome composition and RNA biotypes in sperm samples

To further characterize the RNA composition of sperm samples, RNA biotype distribution was analyzed across bulls and between control and PAM3-treated groups (**Figure 4**). Among the three bulls, mitochondrial ribosomal RNA (mt-rRNA) represented the largest proportion of detected RNA biotypes, accounting for more than 70% of the total annotated reads in all bulls (**Figure 4A**). This proportion was highest in Bull 1, where mt-rRNA accounted for more than 85% of the detected RNA biotypes. Protein-coding RNA represented the second largest category, ranging from approximately 11% to 20%, while unknown RNA accounted for approximately 2% to 3%. When control and PAM3-treated samples were compared within each bull, the overall RNA biotype distribution appeared broadly similar between groups (**Figure 4B**), suggesting that PAM3 treatment did not markedly alter the global proportion of major RNA biotypes.

**Figure 4:**
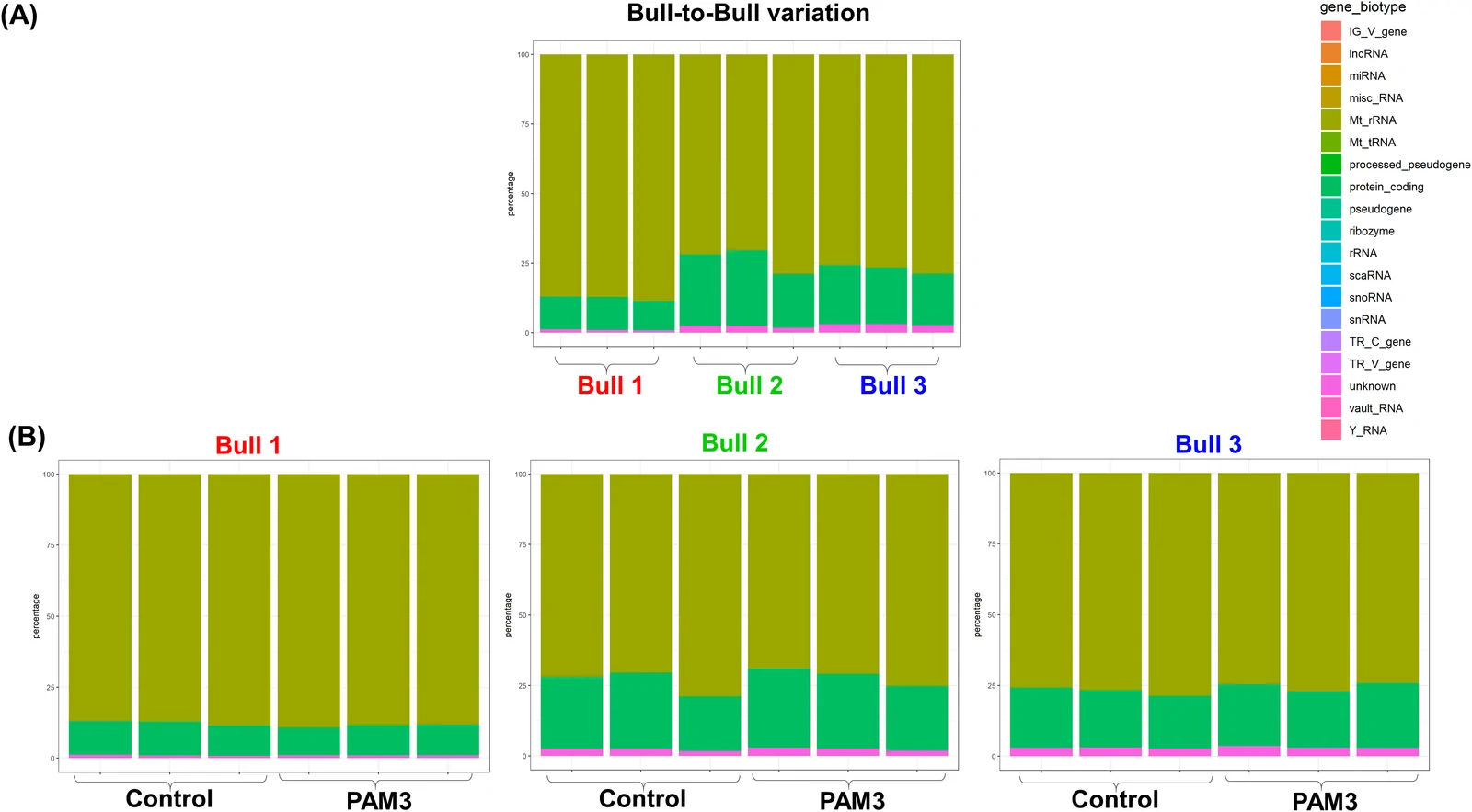
RNA biotype proportion in sperm transcriptomes. **(A)** RNA biotype distribution in sperm samples from three high-fertility Holstein bulls. For comparing three bulls, only the untreated control samples from the three bulls were included. The purpose of this analysis was to assess RNA biotype proportion among bulls independently of PAM3 treatment. Mt-rRNA and protein-coding transcripts represented the major RNA biotypes in all bulls; however, their proportions showed slight variation among individual bulls. **(B)** For comparing control and PAM3, the same control samples were subsequently compared with their corresponding PAM3-treated samples in separate within-bull analyses. RNA biotype distribution in bull sperm after TLR2/1 activation by PAM3 treatment. In all bulls, Mt-rRNA remained the most abundant RNA biotype, and PAM3 treatment did not markedly alter the overall RNA biotype proportion.

To obtain a more detailed view of paternal non-coding RNA composition, the relative proportion of long non-coding RNA (lncRNA) was further examined (**Supplementary Figure 2**). LncRNAs are non-coding transcripts longer than 200 nucleotides and are involved in the regulation of gene expression and several biological processes, including spermatogenesis and sperm function (Joshi and Rajender, 2020). Previous studies have shown that sperm contain diverse RNA species, including mRNAs and lncRNAs, although the functional significance of many sperm lncRNAs remains incompletely understood (Joshi and Rajender, 2020).

Across the three bulls, lncRNAs represented only a very small fraction of the detected RNA biotypes, ranging from below 0.001% to above 0.003% (**Supplementary Figure 2**). This low proportion is consistent with the relatively limited abundance of long non-coding transcripts in mature sperm compared with major RNA categories such as mitochondrial ribosomal RNA and protein-coding RNA (Jodar, 2019). However, low relative abundance does not exclude potential biological relevance, as lncRNAs can contribute to gene regulatory processes (Zhao et al., 2021). When control and PAM3-treated sperm were compared within each bull, PAM3-treated samples showed a higher relative proportion of lncRNA compared with control samples (**Supplementary Figure 2**). In Bull 1, the lncRNA proportion was below 0.001% in control samples and approximately 0.002% after PAM3 treatment. A similar trend was observed in Bull 2, although variation was present within both control and treated groups. In Bull 3, control samples showed lncRNA proportions below approximately 0.0025%, whereas PAM3-treated samples showed higher values, ranging from approximately 0.005% to 0.0075%. These results suggest that PAM3 treatment may be associated with an increased relative proportion of lncRNA in sperm. Because lncRNAs represented only a minor fraction of the detected RNA biotypes, the observed treatment-associated differences should be interpreted cautiously and confirmed using approaches specifically designed for lncRNA detection and validation.

### 3.3 Differential expression analysis in sperm samples

To investigate protein-coding RNA profiles among bulls and to assess the effect of PAM3 treatment on sperm mRNA abundance, differential transcript abundance analysis was performed. Volcano plots were generated to visualize differences in transcript abundance as a function of log2 fold change and FDR (**Figure 5**). Because mature sperm are transcriptionally inactive, these differences were interpreted as changes in the relative abundance of retained sperm transcripts rather than active gene expression (Grunewald et al., 2005).

**Figure 5:**
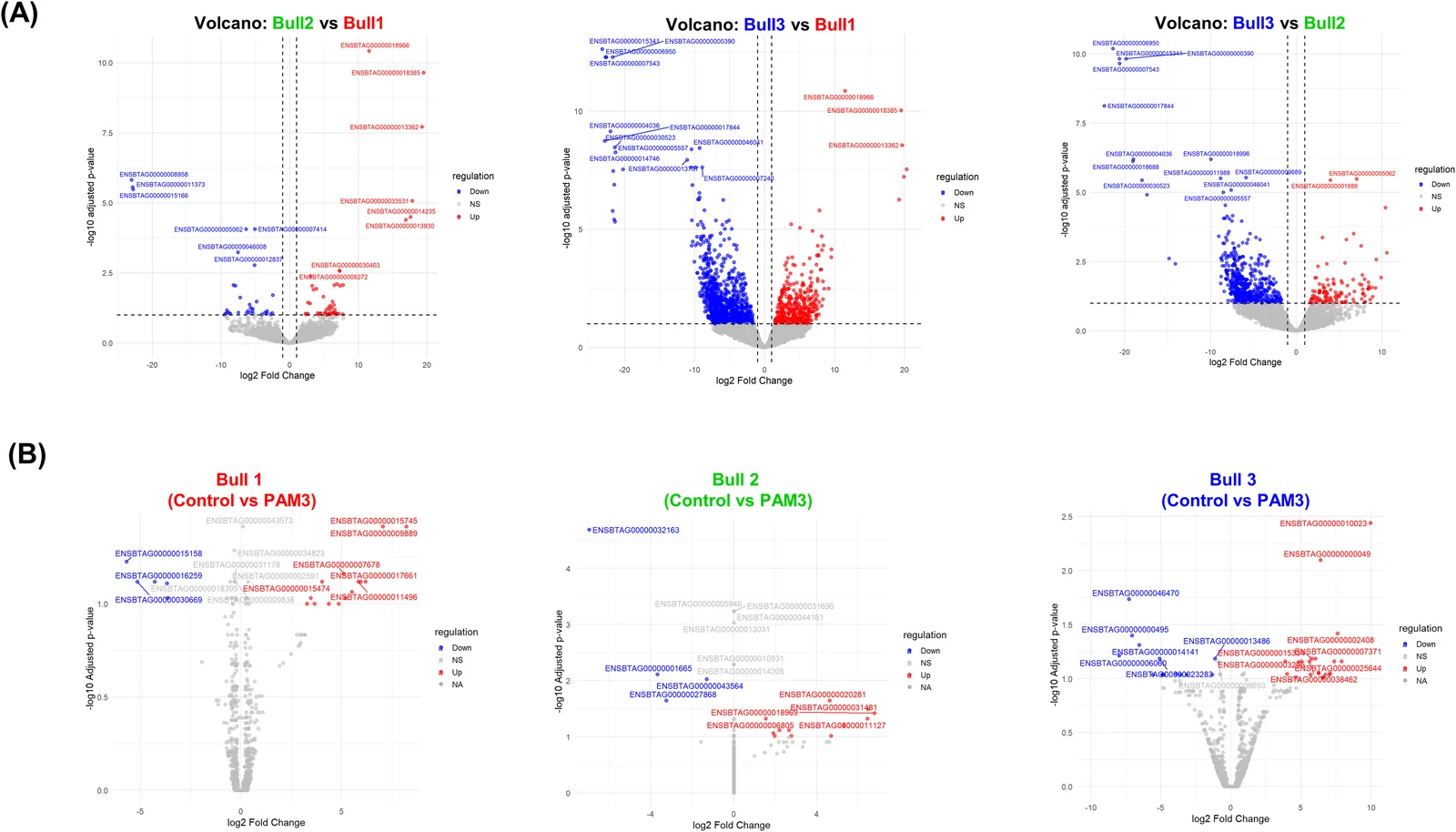
Differentially expressed gene (DEG) analysis using volcano plots. Red and blue dots indicate upregulated and downregulated genes, respectively. **(A)** For comparing three bulls, only the untreated control samples from the three bulls were included. The purpose of this analysis was to assess volcano plot among bulls independently of PAM3 treatment. Volcano plots showing the expression ratios, presented as log2 fold change, and statistical significance of DEGs among the three high-fertility bulls. The results indicate clear transcriptomic differences between each pair of bulls. Notably, Bull 3 showed a greater difference compared with Bulls 1 and 2. **(B)** For comparing control and PAM3, the same control samples were subsequently compared with their corresponding PAM3-treated samples in separate within-bull analyses. Volcano plots showing DEGs between control and TLR2/1-activated sperm within each bull. The results indicate that PAM3 treatment altered the abundance and degradation rate of pre-existing mRNA transcripts in sperm from all bulls.

First, protein-coding transcript profiles were compared among Bulls 1, 2, and 3 to evaluate inter-bull variation (**Figure 5A**). The volcano plots showed clear differences in transcript abundance among bulls. The smallest difference was observed between Bulls 1 and 2, whereas Bull 3 showed a more distinct transcript profile compared with Bulls 1 and 2. These results further support the presence of inter-bull variation in sperm RNA profiles, even among bulls classified as highly fertile.

Next, transcript abundance analysis was performed within each bull to examine the effect of PAM3 treatment on sperm mRNA content (**Figure 5B**). PAM3 treatment was associated with changes in the abundance of a subset of protein-coding transcripts in all bulls. In Bull 1, 17 transcripts showed increased abundance and 15 transcripts showed decreased abundance following PAM3 treatment. In Bull 2, 15 transcripts showed increased abundance, and 12 showed decreased abundance, while in Bull 3, 20 transcripts showed increased abundance and 15 showed decreased abundance (**Table 1**). These findings suggest that PAM3 treatment is associated with bull-specific changes in the abundance of retained sperm mRNA transcripts.

**Table 1:** Increased and decreased abundance of protein-coding transcripts in sperm after TLR2/1 activation.

Bull	Abundance	Protein-coding transcripts
Bull 1	Increased	*RFX2, BRPF3, CD200, TMED2, UBE2V1, RAB3IL1, RIPK2, SRSF11, PLAU, GABPA, TEX36, RANBP ZF, GLTP, ALDOA, SPRED3, UBE2V1, ZNRF2*
	Decreased	*ZC3H10, SSBP2, RPS11, AGGF1, TEC, RRP15, FGGY, PDE4A, SLC9A9, SLC25A24, VWA9, RBFOX2, FAF1, KIAA1217, KIAA1958*
Bull 2	Increased	*SnoRNA, E2F7, UBE2J1, MTSS1, IL2, SPG11, GPR87, TTLL2, ZNF180, SPEN, VPS41, NIN, NUP205, POLR1C, RCBTB1*
	Decreased	*TMEM62, MT-ATP8, PRKAR2A, CCDC146, STAG3, USP13, BEND5, SLC9B1, SSBP1, HYPK, MARK4, NMU*
Bull 3	Increased	*ELP3, CALM1, CCDC77, NAMPT, ANKIB1, STARD3NL, CEP120, GNL3, LEO1, STIL, KLHL10, PPP1R36, CERS4, SCAMP1, TMEM182, ZHX1, TWNK, MAVS, MRPS28, FHAD1*
	Decreased	*UAP1, PRKD3, DGKI, CDRT4, PDE1A, FBLN5, JAML, C12orf66, SPATA4, EFCAB1, TIMM21, RAPGEF1, OTUB1, ONECUT1, TMEM68*

To further compare the transcript-level response across bulls, transcripts belonging to similar gene families were summarized (**Table 2**). Five gene families, including *UBE2*, *SSBP*, *PDE*, *TMEM*, and *ZNF-related* families, showed treatment-associated differences in transcript abundance across bulls. These results suggest that PAM3-mediated TLR2/1 activation may influence the relative abundance of selected protein-coding transcripts in sperm; however, the direction and magnitude of these changes appear to differ among individual bulls.

**Table 2:** Increased and decreased abundance of gene families across all three bulls after TLR2/1 activation.

Gene family	Bull	Gene name	Abundance
UBE2 family ubiquitin conjugating enzyme E2	Bull 1	*UBE2V1*	Increased
	Bull 2	*UBE2J1*	Increased
SSBP family single stranded DNA binding protein	Bull 1	*SSBP2*	Decreased
	Bull 2	*SSBP1*	Decreased
PDE family phosphodiesterase	Bull 1	*PDE4A*	Decreased
	Bull 3	*PDE1A*	Decreased
TMEM genes transmembrane protein	Bull 2	*TMEM62*	Decreased
	Bull 3	*TMEM182*	Increased
	Bull 3	*TMEM68*	Decreased
ZNF-related zinc finger protein	Bull 1	*ZNRF2*	Increased
	Bull 2	*ZNF180*	Increased

To additionally characterize the functional composition of sperm RNA-associated genes, detected genes were classified into major biological categories in control and PAM3-treated sperm samples from each bull (**Supplementary Table 1**). The categories included signaling, mitochondrial/energy metabolism, transport/ion channels, ribosomal/translation-related genes, apoptosis, immune/inflammatory regulation, development/embryo-related genes, and the “other” category comprising genes related to structure, DNA binding, transcriptional regulation, protein modification, unknown function, and other cellular processes. Across all bulls and treatment groups, signaling-related genes represented approximately 10-11% of the classified genes. Mitochondrial/energy-related genes accounted for approximately 2-3%, while transport/ion channel-related genes represented approximately 2-4%. Ribosomal/translation-related genes accounted for approximately 1-2%, with only minor variation among groups. Genes related to immune/inflammatory regulation and development/embryo-associated processes represented smaller proportions, generally below 1%. The remaining genes were classified into the “other” category. Overall, these results indicate that the broad functional distribution of sperm RNA-associated genes was largely similar across bulls and treatment groups, suggesting that PAM3 treatment did not markedly alter the overall functional category profile of detected sperm transcripts.

To assess the potential effects of PAM3 treatment on biological pathways, pre-ranked Gene Set Enrichment Analysis (GSEA) was performed using the complete ranked gene list from the comparison of PAM3-treated and control sperm samples across all bulls (**Supplementary Figure 3**). Several signaling pathways, including those related to immune responses and cell activation, were among the top-ranked GO Biological Process terms and KEGG pathways. However, none remained statistically significant after correction for multiple testing. Therefore, these findings are considered exploratory and could not be interpreted as evidence of significant functional enrichment.

### 3.4 Sperm TLR2/1 activation enhances cleavage and blastocyst rates

To evaluate the functional impact of TLR2/1 signaling on bovine embryo production, we assessed cleavage and blastocyst rates as key indicators of early post-fertilization development and subsequent developmental competence, respectively. When data from all bulls were pooled, PAM3 treatment significantly enhanced both developmental milestones compared with the control group (**Figure 6A**). Specifically, the cleavage rate was significantly higher in the PAM3-treated group (P = 0.015), and a substantial increase was observed in the blastocyst rate (P = 0.0028). Analysis of individual bulls revealed distinct, bull-specific responses to TLR2/1 activation (**Figure 6B**). In Bull 1, PAM3 treatment significantly improved the blastocyst rate compared to the control (P = 0.03), whereas the cleavage rate remained unaffected. Conversely, in Bull 2, PAM3 treatment led to a significant increase in the cleavage rate (P = 0.03), but the improvement in the blastocyst rate did not reach statistical significance. In Bull 3, both cleavage and blastocyst rates exhibited an upward trend following PAM3 treatment, although these differences did not achieve statistical significance. Overall, these findings demonstrate that sperm TLR2/1 activation *via* PAM3 promotes embryonic developmental outcomes. However, the magnitude of the response and the specific developmental stage influenced by the treatment appear to be modulated by individual bull-dependent factors.

**Figure 6:**
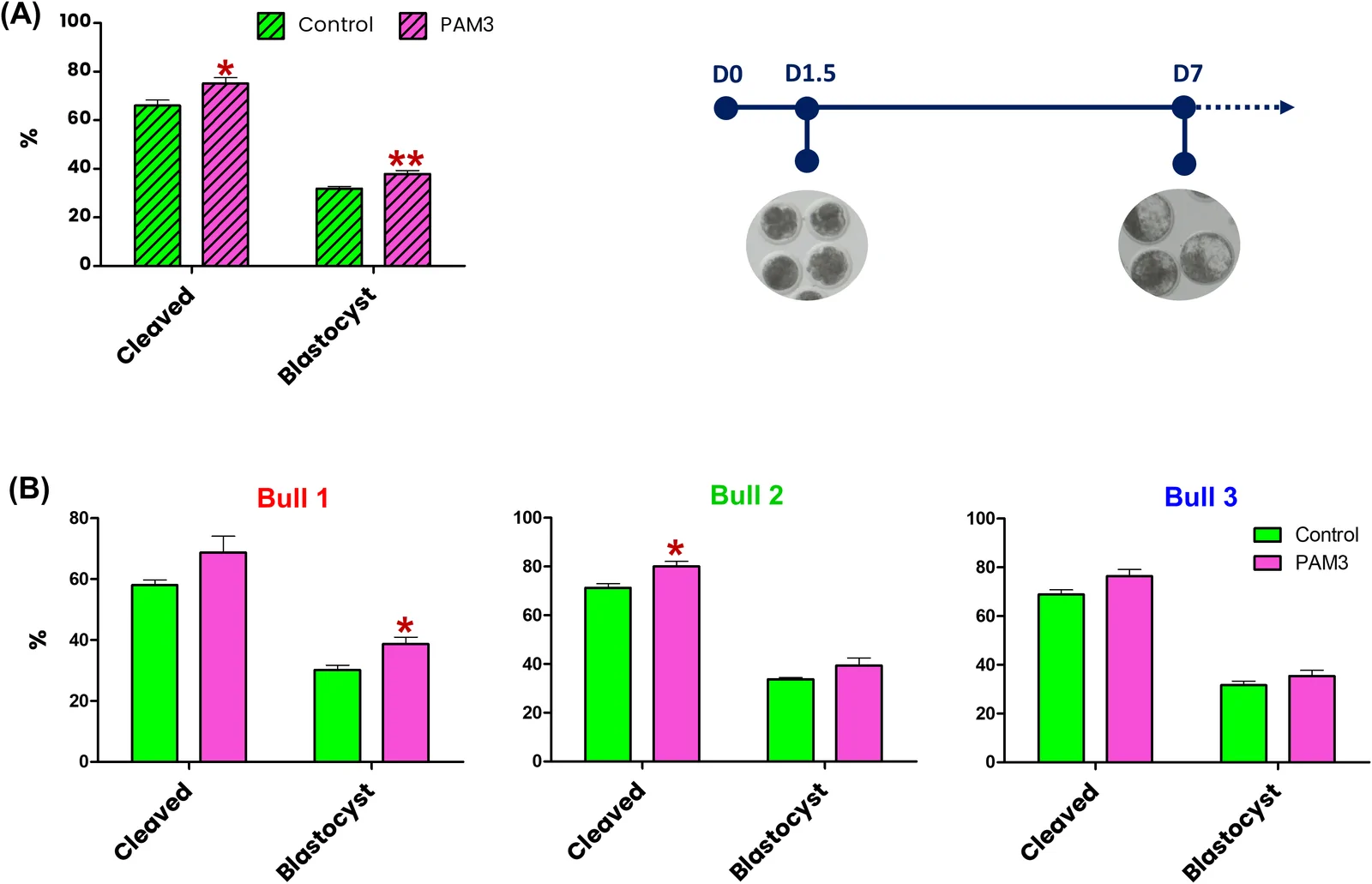
Effect of TLR2/1 activation on embryonic development. Embryonic development was evaluated based on cleavage rate (day 1.5) and blastocyst rate (day 7). Control embryos were derived from untreated sperm, whereas PAM3 embryos were derived from sperm pretreated with PAM3 before IVF. **(A)** Overall effect of PAM3 treatment on embryonic development, with data from all bulls pooled together. **(B)** Effect of PAM3 treatment on embryonic development in each individual bull. Bull 1 (control, n = 361; PAM3, n = 316; 3 replicates), Bull 2 (control, n = 354; PAM3, n = 373; 3 replicates), and Bull 3 (control, n = 323; PAM3, n = 295; 3 replicates).

### 3.5 Limited transcriptomic separation in day 7 embryos across bulls and PAM3 treatment

PCA was performed to evaluate global transcriptomic patterns in day 7 embryo samples and to examine both inter-bull variability and the effect of sperm-PAM3 treatment. PCA was first used to compare embryo transcriptomic profiles among the three high-fertility Holstein bulls (**Figure 7A**). PC1 and PC2 explained 41% and 20% of the total variance, respectively, together accounting for 61% of the variation in the dataset. Samples from the three bulls did not form clearly separated clusters along either PC1 or PC2, suggesting limited inter-bull separation in day 7 embryo transcriptomic profiles.

**Figure 7:**
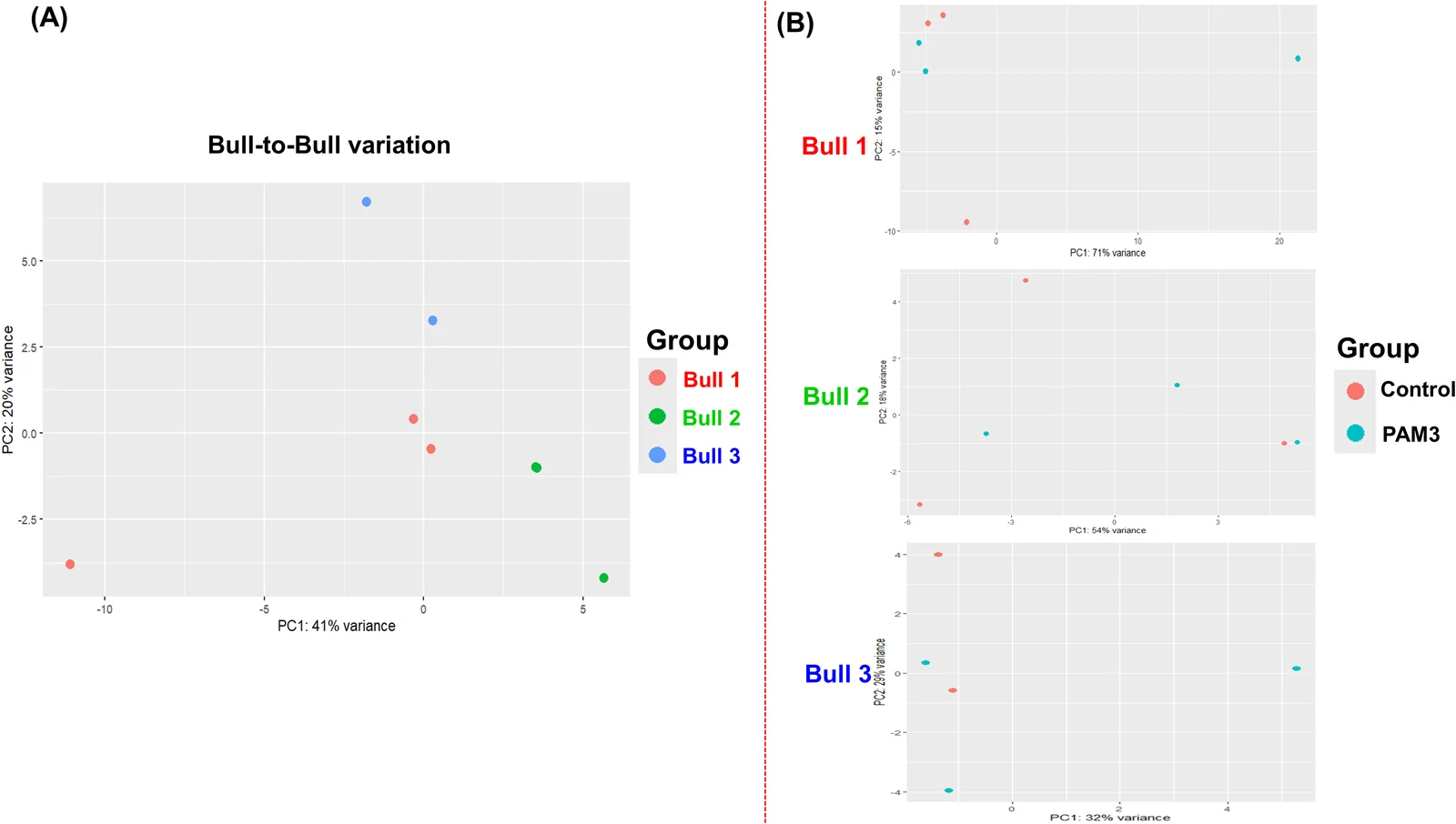
Principal component analysis (PCA) of embryo transcriptomes. **(A)** PCA plot showing transcriptomic variation among embryo samples derived from three high-fertility Holstein bulls. PC1 and PC2 explained 41% and 20% of the total variance, respectively. Although the embryos were derived from different bulls, the PCA plot did not show clear bull-dependent separation in embryo transcriptomic profiles. **(B)** PCA plot showing the effect of sperm TLR2/1 activation by PAM3 treatment on embryo transcriptomes derived from each bull. Sperm PAM3 treatment did not markedly alter the transcriptomic distribution of embryo samples in any bull. For Bull 2 only, a very slight separation was observed along PC2; however, one control replicate did not cluster with the other control samples, and no separation was observed between the control and PAM3-treated samples along PC1.

To further examine the effect of sperm TLR2/1 activation, PCA was performed separately for control and PAM3-group embryo samples within each bull (**Figure 7B**). The first two principal components explained 86%, 72%, and 99% of the total variance in Bulls 1, 2, and 3, respectively, indicating that the PCA captured a substantial proportion of transcriptomic variation within each bull. However, control and PAM3-group embryos did not show clear treatment-dependent clustering in any bull. These results suggest that sperm-PAM3 treatment was not associated with a strong global shift in day 7 embryo transcriptomic profiles, despite its positive effect on embryo developmental outcomes.

### 3.6 Transcriptome composition and RNA biotypes in day 7 embryo samples

To further characterize the RNA composition of day 7 embryo samples, RNA biotype distribution was analyzed across bulls and between control embryos and embryos derived from PAM3-treated sperm (**Figure 8**). Across the three bulls, Mt-rRNA and protein-coding RNA represented the predominant RNA biotypes detected. Mt-rRNA accounted for more than 38% of the total annotated reads, while protein-coding RNA accounted for more than 41% in all bulls (**Figure 8A**). When control embryos and embryos derived from PAM3-treated sperm were compared within each bull, the overall RNA biotype distribution appeared broadly similar between groups (**Figure 8B**), suggesting that sperm-PAM3 treatment did not markedly alter the global proportion of major RNA biotypes in day-7 embryos.

**Figure 8:**
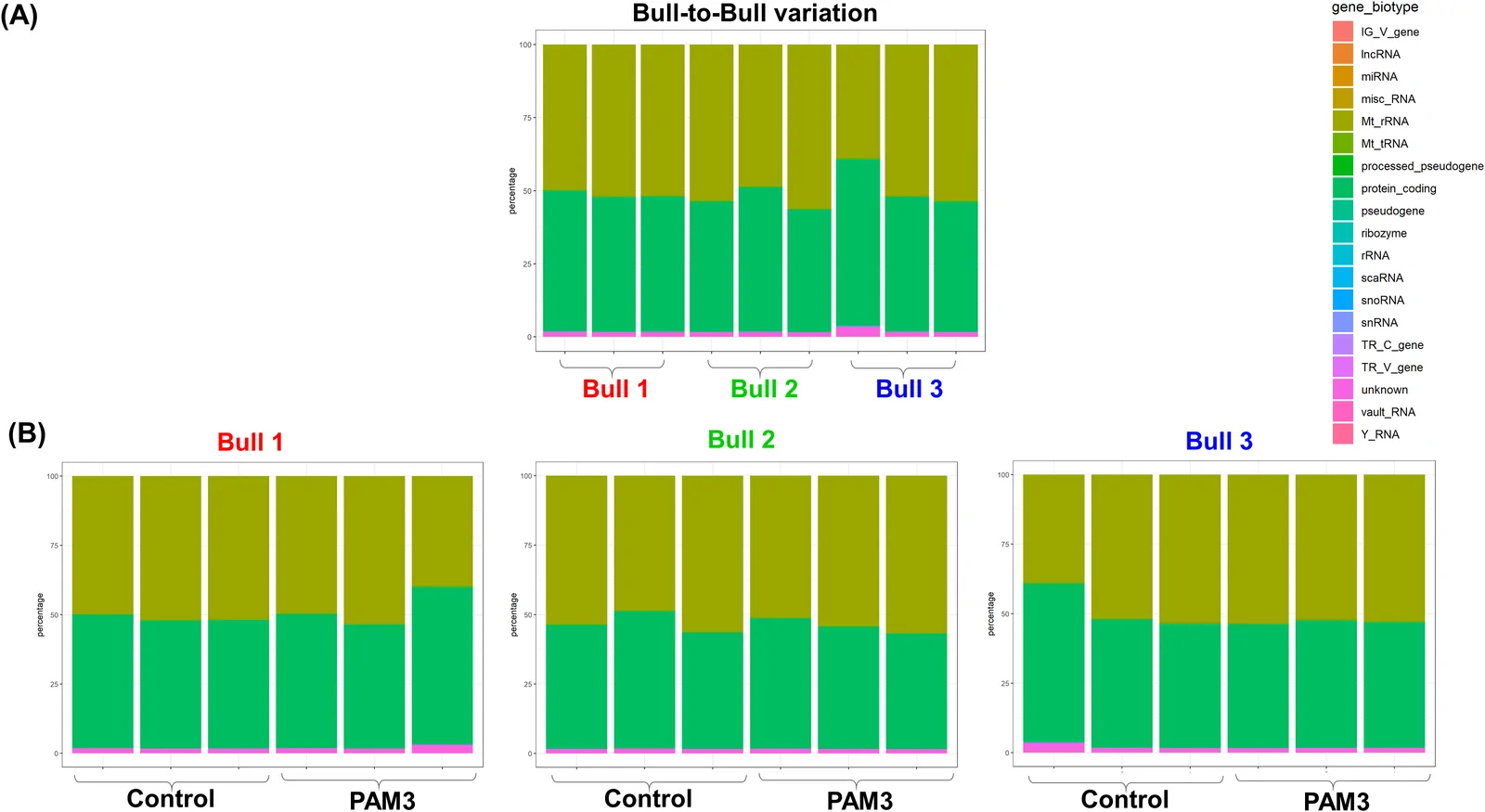
RNA biotype proportion in embryo transcriptomes. **(A)** RNA biotype distribution in embryo samples from three high-fertility Holstein bulls. Mt-rRNA and protein-coding transcripts represented the major RNA biotypes in all bulls; however, their proportions showed slight variation among individual bulls. **(B)** RNA biotype distribution in embryo after sperm TLR2/1 activation by PAM3 treatment. In all bulls, Mt-rRNA and protein-coding remained the most abundant RNA biotypes, and PAM3 treatment did not markedly alter the overall RNA biotype proportion.

To obtain a more detailed view of non-coding RNA composition, the proportion of lncRNA was further examined (**Supplementary Figure 4**). LncRNAs represented a small fraction of the detected RNA biotypes across the three bulls, ranging from below 0.01% to above 0.03%. This proportion was approximately tenfold higher than that observed in sperm samples (**Supplementary Figure 2**). Although lncRNAs accounted for only a minor fraction of the total detected RNA biotypes, they are known to contribute to gene regulatory processes during embryogenesis and early development (Bouckenheimer et al., 2016). When control embryos and embryos derived from PAM3-treated sperm were compared within each bull, no clear treatment-associated increase in the relative proportion of lncRNAs was observed. In Bulls 1, 2, and 3, the lncRNA proportion remained broadly similar between groups, ranging approximately from 0.02% to 0.03% in both control embryos and embryos derived from PAM3-treated sperm. These results suggest that sperm-PAM3 treatment was not associated with a marked change in the global lncRNA proportion in day-7 embryos.

## 4 Discussion

Our findings demonstrate that TLR2/1 signaling is a fundamental modulator of bovine paternal contributions to early development. While previous research established that TLR2/1 activation in sperm enhances fertilization parameters (Ma et al., 2022; Akthar et al., 2023; Hashimoto et al., 2026), the present study suggests a critical molecular link between these cellular changes and improved embryonic outcomes. We show that pre-fertilization stimulation of sperm with a TLR2/1 agonist significantly enhances cleavage and day 7 blastocyst rates. These functional improvements were associated with selective remodeling of the paternal RNA cargo. Specifically, TLR2/1 activation induces shifts in some protein-coding transcripts and long non-coding RNAs across individual bulls, despite inherent inter-bull transcriptomic variation. Regarding the possible causes of this variation and the different developmental responses, differences in TLR2 receptor abundance among individual bulls may contribute to the observed bull-specific responses to PAM3 stimulation. In addition, genetic polymorphisms in bovine TLR2 may affect receptor activity and downstream responses to TLR2/1 activation (Mansouri et al., 2024). However, because TLR2 protein abundance and genetic polymorphisms were not directly assessed in the present study, these explanations remain probable hypotheses that require further investigation. These results highlight the potential of targeting paternal innate immune pathways to optimize bovine embryo production and provide new insights into the complexity of sperm-mediated developmental programming.

A central finding of this study is the temporal decoupling between the altered sperm RNA profile and the stable day 7 embryonic transcriptome. Although PAM3 treatment remodeled the paternal RNA cargo, these differences did not persist as global transcriptomic shifts in the resulting blastocysts. This suggests that the regulatory influence of the modulated paternal RNA cargo is primarily exerted during the earliest stages of development (Graf et al., 2014). In bovine embryos, embryonic genome activation (EGA) begins with a minor wave of early transcriptional activity before the 8-cell stage, followed by major genome activation between the 8- and 16-cell stages, marking the transition from dependence on gamete-derived components to autonomous embryonic transcription (Rabaglino et al., 2023). Consequently, paternal RNAs delivered at fertilization are expected to have their most profound impact on the molecular events preceding and encompassing EGA. We hypothesize that the PAM3-mediated alterations in the sperm transcriptome influence the initial cleavage divisions (e.g., the 2- to 8-cell stages); however, this paternal signature likely becomes weakened by the blastocyst stage as the embryonic genome becomes the dominant driver of the transcriptome. This interpretation is consistent with established models of paternal epigenetic inheritance, which suggest that sperm-borne RNAs can reprogram early embryonic gene expression during the first few mitotic cycles (Tomar et al., 2024; Isacson et al., 2025; Li et al., 2026). Therefore, the improved developmental competence observed in our study likely results from enhanced early-stage stability or more efficient EGA (EGA initiates as early as the one-cell stage (Asami et al., 2022)), rather than a sustained alteration of the steady-state transcriptome at the late blastocyst stage.

Regarding gene expression in sperm, mature sperm are transcriptionally inactive with respect to nuclear DNA, their RNA profile primarily comprises stabilized fragments of spermatogenesis and specific epigenetic carriers (Grunewald et al., 2005). Consequently, the PAM3-induced modifications in sperm transcript abundance likely result from altered RNA stability or selective degradation rather than *de novo* transcription. Crucially, because RNA sequencing measures relative proportions rather than absolute transcript counts, the apparent upregulation of certain nuclear-encoded RNAs may reflect their inherent resistance to ribonuclease activity amid the rapid degradation of more fragile transcripts. Although mitochondrial-derived transcripts could theoretically affect RNA-seq normalization, their abundance did not differ between control and PAM3-treated sperm in our dataset. Therefore, the observed changes in nuclear-derived sperm RNAs are unlikely to be due to mitochondrial transcript changes (Zhu et al., 2019b, Zhu et al., 2019a). We hypothesize that TLR2/1 signaling may trigger intracellular pathways that protect specific transcripts from ribonuclease activity while facilitating the degradation of others. This differential processing could lead to the preservation of essential full-length transcripts or the generation of biologically active, mRNA-derived small RNA fragments. Sperm are known to harbor a diverse repertoire of RNA species, including microRNAs (miRNAs), piwi-interacting RNAs (piRNAs), tRNA-derived small RNAs (tsRNAs), lncRNAs, and mRNAs, many of which are successfully delivered to the ooplasm upon fertilization (González and González, 2025). Previous studies have demonstrated that environmental stressors, such as paternal diet or metabolic state, can remodel the sperm small RNA landscape, subsequently reprogramming embryonic metabolism and developmental trajectories (Isacson et al., 2025). Likewise, PAM3-triggered alterations in the paternal RNA cargo may serve as a transient signaling mechanism that modulates early embryonic programs, even if these signatures are no longer detectable at the blastocyst stage.

Our RNA-seq analysis identified several differentially abundant transcripts in PAM3-treated sperm with established roles in gametogenesis and early embryogenesis. In Bull 1, we observed increased abundance of transcripts such as *RFX2*, *BRPF3*, *CD200*, *TMED2*, *GABPA*, and *PLAU*. Notably, *RFX2* is a master regulator of spermiogenesis; Rfx2-null mice exhibit arrest at the round spermatid stage, resulting in complete infertility (Wu et al., 2016). Furthermore, *TMED2* is essential for the morphogenesis of the embryo and placenta (Jerome-Majewska et al., 2010), while *GABPA* is critical for preimplantation development, with Gabpa-deficient murine embryos failing to reach the blastocyst stage (Liao et al., 2026). Similarly, in Bull 2, PAM3 treatment upregulated transcripts including *E2F7*, *SPEN*, *VPS41*, *NUP205*, and *SSBP1*, several of which are linked to organogenesis or embryonic lethality in knockout models. In Bull 3, treated sperm exhibited higher levels of *NAMPT*, *GNL3* (nucleostemin), *STIL*, *CALM1*, and *KLHL10*. Many of these factors are known determinants of early embryo viability. For instance, *NAMPT* is a rate-limiting enzyme in NAD+ biosynthesis, and its deficiency is associated with compromised developmental potential (Wang et al., 2021). *GNL3* is required for cellular proliferation in the early embryo, with null embryos arresting at the morula stage (Schettini et al., 2024), while *STIL* is indispensable for centriole biogenesis and early mitotic stability (David et al., 2014). Although the current dataset did not permit a formal pathway enrichment analysis due to the selective nature of the transcriptomic changes, the clustering of these specifically regulated genes suggests that TLR2/1 activation modulates a subset of the paternal RNA cargo involved in gamete function and early embryonic viability.

Collectively, these findings support the hypothesis that TLR2/1 activation in sperm modulates the paternal contribution to the zygote, likely by altering early developmental signals. Sperm-borne RNAs, including both coding and non-coding species, function as critical epigenetic vectors that influence the earliest stages of embryogenesis (Aliakbari et al., 2021). For instance, numerous sperm-derived miRNAs and tRNA-derived small RNAs (tsRNAs) are delivered to the ooplasm at fertilization, where they actively regulate the first waves of zygotic gene expression (Hitit et al., 2024; Tomar et al., 2024). Similarly, the differentially abundant mRNA profiles identified in this study, including transcripts such as *RFX2*, *PLAU*, and *TMED2*, may be transferred to the oocyte to modulate initial transcriptional or signaling events.

The molecular shifts induced by PAM3 in sperm likely involve the activation of conserved intracellular cascades, such as Ca^2+^ signaling and autophagy. Previous work has demonstrated that TLR2/1 stimulation in sperm triggers an influx of intracellular Ca^2+^, promoting hyperactivation and acrosomal readiness, while similar TLR2-mediated Ca^2+^ dynamics in embryos have been shown to induce autophagy and mitigate apoptosis (Ma et al., 2022, Ma et al., 2025). While our current transcriptomic data do not directly measure these protein-level processes, TLR2/1 signaling may initiate cascades involving adaptor kinases such as RIPK2, whose transcript abundance was increased in Bull 1; these pathways may remodel the sperm RNA cargo or epigenome. Such modifications could enhance subsequent embryonic development.

In this context, we previously defined embryonic quality as a multifaceted concept encompassing the embryo’s ability to reach advanced stages with appropriate timing, maintain cellular health, and possess efficient regulatory mechanisms such as modulated Ca^2+^ signaling and autophagic flux necessary for successful implantation (Ma et al., 2025). Our prior research in Japanese Black cattle demonstrated that sperm-TLR2 activation significantly enhances these specific quality indicators, yielding results comparable to those achieved when the TLR2 pathway is activated directly at the 4-cell stage (Ma et al., 2022; Hashimoto et al., 2026). Although the present study focused on cleavage and blastocyst rates in Holstein bulls, the observed improvements in these developmental milestones likely reflect a similar enhancement of underlying cellular quality. Notably, the lack of transcriptomic divergence on day 7 in the current study suggests that these paternal influences are transient yet impactful. They likely provide transient support to early developmental programs or facilitate more efficient epigenetic reprogramming during EGA (Graf et al., 2014), allowing a higher percentage of embryos to survive the transition to autonomous genomic control without requiring a sustained alteration of the steady-state transcriptome at the blastocyst stage.

Despite the significant findings presented here, several limitations should be considered. By profiling the transcriptome at the day 7 blastocyst stage, we may have missed the primary temporal window in which paternal RNAs exert their most significant regulatory effects. Future investigations should target the 2-cell, 4-cell, and morula stages to capture transient alterations in gene expression or epigenetic signatures resulting from PAM3-treated sperm. Utilizing high-sensitivity techniques such as single-embryo RNA-seq or targeted qPCR at these pre-implantation stages would clarify whether paternal transcripts persist or selectively modulate early embryonic transcription (Ferreux et al., 2026). Further, while this study focused on the RNA cargo, sperm also deliver a complex set of other epigenetic factors, including DNA methylation patterns, post-translational histone modifications, and retained protamines, that collectively influence developmental paths (Gold et al., 2018). It is possible that innate immune stimulation *via* TLR2/1 modifies these marks; for instance, signaling cascades are known to influence chromatin accessibility and small RNA biogenesis. Therefore, future research should adopt a multi-omics approach, incorporating epigenomic profiling of sperm (e.g., ATAC-seq, bisulfite sequencing) alongside assessments of embryonic epigenetic reprogramming (Hernandez Mora et al., 2023). Finally, although the use of three high-fertility bulls provided a strong initial model, validation across a larger unit of males and under varied *in vitro* culture conditions is necessary to generalize these conclusions. It remains a priority for future studies to confirm that PAM3-mediated modulation does not induce deleterious off-target effects, such as abnormal immune activation or compromised long-term health in the resulting offspring.

A further limitation of this study is the relatively small number of bulls included in the transcriptomic analysis. Although three biological replicates were examined for each of the three high‑fertility bulls, the limited sample size reduces the statistical power to assess inter‑individual variation and constrains the broader generalizability of the results. This work was intentionally designed as a controlled proof‑of‑concept study, focusing mainly on within‑bull comparisons between control and PAM3‑treated samples while offering an initial view of variation across bulls. The marked bull‑specific differences observed in baseline sperm RNA profiles and in transcriptomic responses to PAM3 highlight the substantial influence of individual biological variability. These findings should therefore be interpreted with appropriate caution and validated in future studies that include a larger and more genetically diverse population of bulls. Another limitation of this study is the lack of independent validation of the RNA-seq findings by qRT-PCR. The study was designed as an initial exploratory analysis to assess overall changes in sperm RNA profiles following TLR2/1 activation, rather than to validate specific transcripts as biomarkers or mechanistic targets. Therefore, the transcript-level findings should be considered preliminary and hypothesis-generating. Future studies should validate selected transcripts using qRT-PCR or other targeted approaches in independent samples and in a larger group of bulls.

In conclusion, this study provides the first evidence that TLR2/1 activation in bovine sperm *via* PAM3 selectively remodels the paternal RNA cargo and significantly enhances IVF success. Notably, these functional improvements in developmental competence, specifically increased cleavage and blastocyst rates, occur without inducing lasting global transcriptomic alterations in the resulting day 7 blastocysts (**Figure 9**). Our data suggest that the primary regulatory impact of PAM3 treatment is concentrated within the pre-implantation developmental window, potentially mediated by the delivery of specific protein-coding and non-coding RNAs from the sperm to the oocyte. These findings align with the established role of paternal RNAs as epigenetic regulators of zygotic gene expression and embryonic viability (Aliakbari et al., 2021; Hitit et al., 2024; Tomar et al., 2024). Continued investigation into pre-implantation developmental milestones, specifically 2-, 4-, 8-, 16-, and 32-cell stages, as well as the morula and its associated epigenetic mechanisms will be essential to clarify how TLR2/1 signaling can be optimally connected to improve cattle IVP and to determine whether these effects outline a physiological paternal enhancement of embryonic competence.

**Figure 9:**
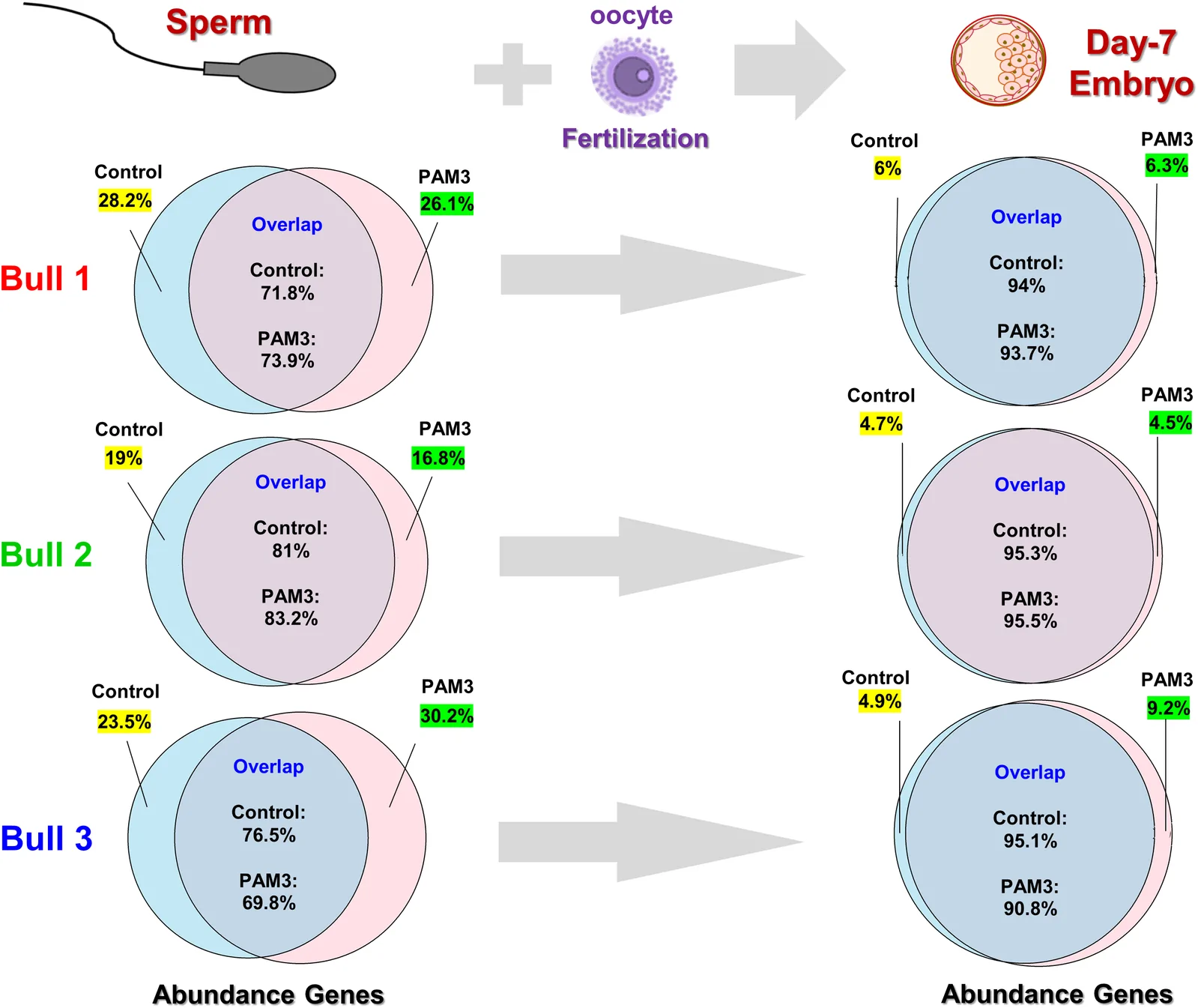
Overall findings. Based on our findings, activation of sperm TLR2/1 by PAM3 may alter the paternal transcriptomic profile. Across all bulls, approximately 20% of sperm RNAs were specific to either the control or PAM3-treated group, whereas approximately 70% were shared between the two groups. For example, in Bull 1 sperm, 26.1% of total RNAs detected in the PAM3-treated group were not abundant in the control group, while 28.2% of total RNAs detected in the control group were not abundant in the PAM3-treated group. These alterations may include decreased abundance of pre-existing sperm mRNAs. Because sperm are transcriptionally inactive, these changes are unlikely result from new transcription. Instead, they may reflect selective stabilization, degradation, or fragmentation of existing mRNA transcripts following TLR2/1 signaling. Although paternal RNA profiles were altered in sperm, these changes did not appear to persist in day-7 embryos. For example, in embryos derived from Bull 1, only approximately 6% of RNAs were specific to either the PAM3 or control group, while more than 94% were shared between the two groups. These findings suggest that PAM3-induced alterations in paternal sperm RNA are not maintained at the transcriptomic level in day-7 embryos.

## Data Availability

The datasets presented in this study can be found in online repositories. The names of the repository/repositories and accession number(s) can be found below: https://www.ncbi.nlm.nih.gov/, GSE334146.
