# Supplementary material for: Sperm TLR2/1 activation modulates the paternal RNA cargo and enhances bovine embryonic development
Source: Front Physiol. 2026 Sep 23;17:1906057. doi: 10.3389/fphys.2026.1906057 (PMC13642134; doi:10.3389/fphys.2026.1906057)
Supplement: Supplementary file 1 [file DataSheet1.docx]

**Sperm TLR2/1 activation modulates the paternal RNA cargo and enhances bovine embryonic development**

**Alireza Mansouri^1#^*, Ihshan Akthar^1#^, Taiyo Hashimoto^1^, Kazuya Kusama^2^, Kazuhiko Imakawa^3^, Masayuki Shimada^4^, Akio Miyamoto^1^***

^1^Global Agromedicine Research Center (GAMRC), Obihiro University of Agriculture and Veterinary Medicine, Obihiro, Japan

^2^Department of Endocrine Pharmacology, Tokyo University of Pharmacy and Life Sciences, Tokyo, Japan

^3^Research Institute of Agriculture, Tokai University, Kumamoto, Japan

^4^Graduate School of Integrated Sciences for Life, Hiroshima University, Higashi-Hiroshima, Japan

**^#^**These authors contributed equally to this work and share first authorship

^*^Correspondence

Alireza Mansouri

alireza_mansouri89@yahoo.com

Akio Miyamoto

akiomiya@obihiro.ac.jp

TEL +81-155-49-5416/FAX +81-155-49-5593

**Supplementary Table 1.** Major biological categories in control and PAM3-treated sperm samples from each bull.

| **Category** | **Bull1 Control** | **Bull1 PAM3** | **Bull2 Control** | **Bull2 PAM3** | **Bull3 Control** | **Bull3 PAM3** |
| --- | --- | --- | --- | --- | --- | --- |
| **Other** | 81.02 | 80.78 | 81.71 | 81.28 | 81.43 | 80.85 |
| **Signaling** | 10.6 | 11.53 | 11.09 | 10.53 | 11.14 | 10.51 |
| **Mitochondrial/Energy** | 3.12 | 2.31 | 2.46 | 2.63 | 2.25 | 2.22 |
| **Transport/Ion Channel** | 2.42 | 2.84 | 2.92 | 3.08 | 2.5 | 3.5 |
| **Ribosomal/Translation** | 1.66 | 1.84 | 0.84 | 0.98 | 1.64 | 1.79 |
| **Apoptosis** | 0.62 | 0.38 | 0.45 | 0.75 | 0.49 | 0.51 |
| **Immune/Inflammation** | 0.42 | 0.15 | 0.32 | 0.45 | 0.3 | 0.34 |
| **Development/Embryo** | 0.14 | 0.15 | 0.19 | 0.3 | 0.24 | 0.26 |
| **Total** | 100 | 100 | 100 | 100 | 100 | 100 |

**Supplementary Figure 1. Principal component analysis (PCA) of sperm transcriptomes.** PCA plot showing transcriptomic variation among control and PAM3 treatment sperm samples from three high-fertility Holstein bulls.

**Supplementary Figure 2. Long non-coding RNA (lncRNA) profiles in sperm transcriptomes.** lncRNA profiles in sperm samples from all bulls and following TLR2/1 activation by PAM3 treatment. The figure shows the distribution of sperm lncRNA transcripts among individual bulls and compares lncRNA patterns between control and PAM3-treated sperm. The one-way ANOVA comparing lncRNA abundance among the three bulls showed a significant difference among bulls p = 0.0008. This analysis evaluates the effect of bull identity rather than the effect of PAM3 treatment. For the paired comparisons between control and PAM3-treated samples within each bull, the paired t-test results were as follows. Bull 1: p = 0.037, indicating a significant difference. Bull 2: p = 0.954, indicating no significant difference. Bull 3: p = 0.279, indicating no significant difference.

**Supplementary Figure 3. KEGG pathway and GO enrichment analyses of bull sperm under control and PAM3-treated conditions.** None of the GO terms or KEGG pathways remained statistically significant after Benjamini-Hochberg correction for multiple testing.

**Supplementary Figure 4. Long non-coding RNA (lncRNA) profiles in embryo transcriptomes.** lncRNA profiles in embryo samples from all bulls and following sperm TLR2/1 activation by PAM3 treatment. The figure shows the distribution of embryo lncRNA transcripts among individual bulls and compares lncRNA patterns between control and PAM3-treated sperm.
